# Epidemiological trends of adrenalectomies in Brazil: A cohort-based study of the Brazilian public health system

**DOI:** 10.20945/2359-4292-2025-0116

**Published:** 2025-08-18

**Authors:** José Gustavo Olijnyk, Maysa Tayane Santos Silva, Leandro Totti Cavazzola, Mauro Antônio Czepielewski

**Affiliations:** 1 Hospital Nossa Senhora da Conceição, Serviço de Endocrinologia Clínica e Cirúrgica, Porto Alegre, RS, Brasil; 2 Hospital Militar de Área de Porto Alegre, Serviço de Cirurgia, Porto Alegre, RS, Brasil; 3 Universidade Federal do Rio Grande do Sul, Faculdade de Medicina, Porto Alegre, RS, Brasil; 4 Universidade Federal do Rio Grande do Sul, Departamento de Cirurgia, Pós-graduação em Ciências Cirúrgicas - Cirurgia Minimamente Invasiva, Porto Alegre, RS, Brasil; 5 Universidade Federal do Rio Grande do Sul, Faculdade de Medicina, Pós-graduação em Ciências Médicas - Endocrinologia, Porto Alegre, RS, Brasil

**Keywords:** Adrenalectomy, public Health, Brazil, retrospective cohort study

## Abstract

**Objective:**

Population-based data on the surgical treatment of adrenal diseases in Brazil remain limited. Therefore, this retrospective cohort study aimed to characterize patients treated within the Brazilian public health system who underwent adrenalectomy over the past 15 years.

**Materials and methods:**

Records of adrenalectomies from the *Sistema Único de Saúde* (Datasus) database were analyzed from January 2008 to December 2022. Descriptive demographic information was evaluated using incidence rates. Clinical, therapeutic, and mortality data were compared according to hospital procedure volume stratification.

**Results:**

Over the study period, there was a 49.6% increase in procedures, totaling 6,771 adrenalectomies, with high-volume hospitals performing 62.3% of the cases. Most patients were female (65.5%), white (52%), and had a median age of 48 years. Oncological adrenalectomies increased by 154%, particularly in the southern region of Brazil. Although an overall reduction in in-hospital mortality rates was observed, a higher risk persisted in low-volume centers for both oncological (OR: 2.75; 95% CI: 1.53-4.93; p < 0.01) and non-oncological surgeries (OR: 6.60; 95% CI: 3.98-10.96; p < 0.01).

**Conclusion:**

Given the increasing number of adrenalectomies performed within the Brazilian public health system and the likely continuation of this trend, health policies should prioritize referral to high-volume centers equipped with advanced techniques and appropriate infrastructure.

## INTRODUCTION

The advent of minimally invasive adrenalectomy techniques in the 1990s led to a marked reduction in postoperative morbidity (^1^). To prevent overtreatment of non-functioning benign adenomas or undertreatment of malignant tumors, criteria for surgical intervention have been continuously revised and refined based on the secretory and phenotypic characteristics of tumors (^2^-^4^). Nonetheless, North American data indicate a nearly 45% increase in adrenalectomy procedures over the past 20 years (^5^).

To date, no epidemiological studies have comprehensively evaluated the incidence of adrenalectomies across the entire Brazilian territory, as determined by a search of Medline/PubMed, LILACS, and Embase using the MeSH terms “adrenalectomy” and “Brazil.”

Available information regarding adrenal surgery is limited to a few restricted regions (^6^,^7^). Utilizing the Brazilian public health system database, we aimed to assess the characteristics of surgical treatment for adrenal diseases and to understand potential epidemiological changes.

By assessing the number of adrenalectomies categorized according to the extent of surgery, we aimed to identify the spectrum of adrenal diseases prompting these interventions. Our hypothesis is that unilateral adrenalectomies would have been performed more frequently, as this approach is more commonly indicated for adrenal diseases with a benign phenotype. In contrast, bilateral surgeries would likely comprise a smaller proportion, typically associated with hereditary diseases and bilateral macronodular disease. Adrenalectomies performed for oncological indications, including the treatment of primary carcinoma and metastases from various sites, would reflect their prevalence and evolving trends within the cohort.

Additionally, the geographic distribution of surgeries across Brazilian regions may provide information about the origin of patients, the reference hospitals at which they were treated, and whether the annual surgical volume is associated with differences in outcomes.

## MATERIALS AND METHODS

A retrospective cohort study was conducted on patients who underwent adrenalectomy in Brazilian public hospitals from January 2008 to December 2022. Data were extracted from hospital admission authorizations (known as “reduced AIHs,” or *AIH reduzidas* in Brazilian Portuguese) encompassing all public hospitals in the 27 federative units of Brazil and registered with the National Registry of Health Establishments, a Ministry of Health system used to identify procedures performed during hospitalization.

Additionally, information was collected on sex, race (self-reported), and age group (years: <1, 1-4, 5-14, 25-34, 35-44, 45-54, 55-64, and ≥65), as well as the place of hospitalization and patient residence (both by region of the country), length of hospital stay (days), total costs, intensive care unit (ICU) admissions, and deaths.

Files from the *Sistema Único de Saúde* (SUS) IT Department (Datasus) website and the TabWin415 software were used to tabulate the information, with data accessed up to February 20, 2023 (^8^).

### Statistical analysis

The study period was divided into three five-year intervals, and evolutionary trends were compared using percentage variation. Population data and maps of Brazil were retrieved from the Brazilian Institute of Geography and Statistics website (^9^,^10^) to generate the incidence calculations adjusted to the median population of each interval (194,890,682 inhabitants in 2010, 203,475,683 in 2015, and 227,925,692 in 2020), and the graduated geographic distribution of the residence locations of patients undergoing oncological adrenalectomy (QGIS 3.30 program). Associations between variables and surgical procedures were evaluated using odds ratios (OR), 95% confidence intervals (95% CI), and *p* < 0.05 to determine statistical significance. Hospital surgical volume was stratified and compared with mortality rates using the Kruskal-Wallis test. Two cutoff points were established: six or more adrenalectomies per year and the mortality rate of hospitals performing six adrenalectomies per year was compared with those performing seven or more. Statistical analysis was performed using the IBM SPSS Statistics 20.0.

### Ethical aspects

The Datasus database is publicly available and contains aggregated, anonymized information, with no possibility of direct identification or re-identification of individuals. According to Brazilian National Health Council resolutions (nos. 510/16 and 674/2022) (^11^,^12^), research using databases in which individuals are not identified does not require registration or assessment by an ethics committee.

## RESULTS

Between 2008 and 2022, 468 Brazilian public hospitals performed 6,771 adrenalectomies at a total cost of US$ 3,634,593.10, of which US$ 2,487,641.62 was allocated to oncological surgical treatment (^13^). The demographic data are summarized in **Table 1.** Female patients were predominant (65.5%), with a median age of 48 years. Most patients were reported to be white individuals (52%), followed by those of brown, black, and other ethnicities. There were no records of treatment for Indigenous people, and in 16.6% of the cases, race was not informed. The most commonly performed procedure was unilateral adrenalectomy (51.1%), while oncological surgery accounted for 44.3% and bilateral adrenalectomy for 4.5% of cases.

**Table 1:** Demographic characteristics of 6,771 adrenalectomies (2008-2022)

	Unilateral	Bilateral	Oncological
Total Cases N (%)	3,461	310	3,000
Sex Male Female	1,052 (30.39) 2,409 (69.60)	89 (28.70) 221 (71.29)	1,192 (39.73) 1,808 (60.26)
Age Range < 1 1-4 5-14 15-24 25-34 35-44 45-54 55-64 ≥ 65	46 (1.32) 126 (3.64) 105 (3.03) 184 (5.31) 408 (11.78) 611 (17.65) 775 (22.39) 771(22.27) 435 (12.56)	3 (0.96) 8 (2.58) 20 (6.45) 36 (11.61) 54 (17.41) 56 (18.06) 62 (0.20) 51 (16.45) 20 (6.45)	100 (3.3) 345 (11.5) 162 (5.4) 112 (3.7) 223 (7.4) 352 (11.7) 511 (17.0) 690 (23.0) 505 (16.8)
Race White Black Brown Yellow Not informed	1,722 (49.75) 166 (4.79) 864 (24.96) 40 (1.15) 669 (19.32)	186 (60.0) 6 (1.93) 65 (20.96) 1 (0.32) 52 (16.77)	1,619 (53.96) 94 (3.13) 855 (28.50) 27 (0.90) 405 (13.50)

Source: Ministry of Health - Brazil.

Adrenalectomies increased in general by 49.6%, especially in oncology (**Table 2**). In contrast, the number of non-oncological adrenalectomies remained stable during the study period. The in-hospital mortality rate varied at 0.7%-2.3%, being higher among oncological treatments (2.03% versus 0.84% for non-oncological procedures), although a negative percentage variation was observed in all surgical modalities by the end of the cohort. Admissions to the ICU showed an increasing trend.

**Table 2:** Evolutionary trend of hospital characteristics of adrenalectomies and Brazilian regions

	2008-2012	2013-2017	2018-2022	∆%
**Oncological N (%)**	564	1,003	1,433	+154
Deaths	13 (2.30)	20 (1.99)	28 (1.95)	-15.2
ICU admissions	245 (43.4)	462 (46)	726 (50.6)	+16.5
Non-oncological^*^ N (%)	1,218	1,319	1,234	+1.31
Deaths	11 (0.9)	12 (0.9)	9 (0.7)	-22.2
ICU admissions	407 (33.4)	516 (39.1)	516 (41.8)	+25.1
**Hospitalization/Residence†** North Northeast Central West Southeast South	35 / 46 262 / 269 106 / 890 914 / 468 465 / 109	53 / 61 329 / 333 131 / 1279 1321 / 493 488 / 156	102 / 117 390 / 406 153 / 1396 1455 / 574 567 / 174	

* Grouped unilateral and bilateral adrenalectomy; † Location of hospitalization/Region of residence. Source: Ministry of Health - Brazil.

Analysis of the geographic distribution of surgeries across Brazilian regions (**Table 3**) revealed that 76.8% were performed in southeastern and southern Brazil (54.4 and 22.4%, respectively). The northeastern region accounted for 14.4%, followed by the central-western (5.7%) and northern regions (2.8%). Although there was an overall increase in adrenalectomies nationwide over fifteen years, this did not always correspond to the patient’s place of residence relative to the regions where surgeries were performed.

**Table 3:** Brazilian hospitals (SUS) with high volume in adrenalectomies (7)

Hospital	State	$\bar{x}$
1. Hospital das Clínicas - FMUSP 2. Fundação Pio XII Barretos 3. Hospital de Clínicas - UFRGS 4. Hospital das Clínicas de Ribeirão Preto 5. Santa Casa de São Paulo 6. Hospital Amaral Carvalho 7. Hospital Euryclides de Jesus Zerbini 8. Hospital Nossa Senhora da Conceição 9. INCA Hospital do Câncer 10. Santa Casa de Belo Horizonte 11. Hospital de Base de São José do Rio Preto 12. Santa Casa de Misericórdia de Porto Alegre 13. Hospital Santa Marcelina 14. Instituto do Câncer 15. Hospital de Clínicas - UFPR 16. Hospital das Clínicas - UNICAMP 17. Instituto Hospital de Base 18. Hospital de Câncer de Goiânia 19. A C Camargo Cancer Center 20. Hospital de Clínicas de Uberlândia 21. Hospital Clemente Fraga Filho - UFRJ 22. Hospital Infantil Pequeno Príncipe 23. CEONC 24. Hospital Bom Samaritano 25. Hospital São Lucas da PUCRS 26. Hospital das Clínicas - UFMG	São Paulo São Paulo Rio Grande do Sul São Paulo São Paulo São Paulo São Paulo Rio Grande do Sul Rio de Janeiro Minas Gerais São Paulo Rio Grande do Sul São Paulo São Paulo Paraná São Paulo Distrito Federal Goiás São Paulo Minas Gerais Rio de Janeiro Paraná Paraná Minas Gerais Rio Grande do Sul Minas Gerais	46.80 22.20 16.93 16.80 16.20 11.73 10.67 9.33 9.07 8.73 8.47 8.33 7.93 7.87 7.73 7.67 6.80 6.80 6.73 6.53 6.53 6.47 6.40 6.27 6.20 6.13

$\bar{x}$ , annual mean of adrenalectomies (2008 - 2022). Source: Ministry of Health - Brazil.

Northern Brazil, despite representing a smaller proportion, showed the greatest increase in hospitalizations, followed by southeastern Brazil, with the second largest positive variation. Although the southern region presented the lowest increase, it exhibited the highest number of cases per million inhabitants (4.95 cases/million), followed by the southeastern (3.49 cases/million), central-western (2.4), northeastern (1.76), and northern regions (1.27).

**Figure 1:**
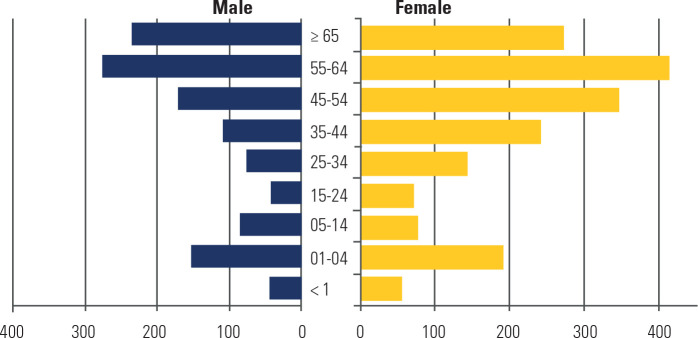
Age distribution of oncological adrenalectomies (2008-2022).

**Figure 2:**
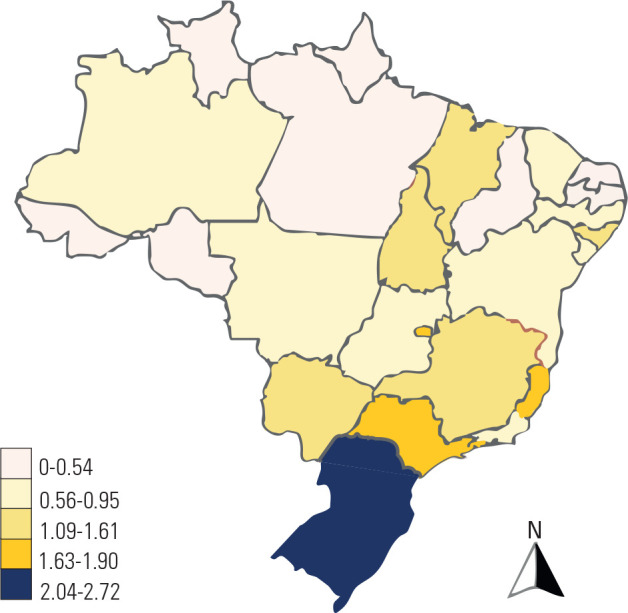
Oncological adrenalectomy per 1,000,000 inhabitants according to place of residence (2018-2022).

The age distribution for the incidence of oncological adrenalectomies showed two peaks (**Figure 1**): one during early childhood (1-4 years) and another in adulthood (55-64 years). In the last five years, the southern region registered the highest incidence among residents aged up to four years (3.2 cases per million in males and 5.6 cases per million in females) (**Figure 2**). In the second age peak, the incidence was 8.8 cases per million in males and 11 per million in females.

Annual mean. Source: Ministry of Health - Brazil.

**Figure 3:**
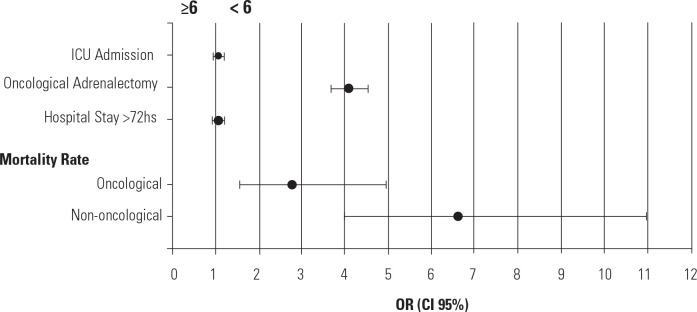
Comparison of factors between hospitals with high and low surgical volume (annual mean of adrenalectomies, 2008-2022).

Among the 26 hospitals with a high volume of adrenal surgeries (5.5%, all teaching hospitals), 4,220 operations were performed (62.3% of all adrenalectomies). Hospitals performing one procedure per year comprised 70.9% (n = 332). Differences in outcomes were observed among hospitals performing six or more cases per year (**Figure 3**).

Regarding oncological adrenalectomies, the likelihood of the procedure being performed in a low-volume center was higher (OR: 4.06; 95% CI: 3.66-4.51; *p* < 0.01), as was the probability of in-hospital mortality (OR: 2.75; 95% CI: 1.53-4.93; *p* < 0.01). For non-oncological surgeries, this difference was even more pronounced, with the risk more than six times higher in low-volume hospitals (OR: 6.60; 95% CI: 3.98-10.96*; p* < 0.01). In-hospital deaths were also associated with patients over 60 years of age (OR: 3.38; 95% CI: 1.48-7.71; *p* < 0.01). No difference in mortality was detected between hospitals performing six adrenalectomies per year and those performing seven or more cases (OR: 0.41; 95% CI: 0.57; *p* = 0.55).

There was no significant difference in prolonged hospitalization (>72 hours) between lowand high-volume centers (OR: 1.05; 95% CI: 0.92-1.19; *p* = 0.44). The same was observed for ICU admissions (OR: 1.05; 95% CI: 0.94-1.18; *p* = 0.32).

## DISCUSSION

During the fifteen-year study period, the annual average number of adrenalectomies was 452. Although this figure does not include procedures performed in the private sector, it is representative, as it covers 71.5% of the Brazilian population (^14^). The 49.6% increase in the number of procedures parallels the trend observed in the United States over the past two decades (^5^), where the annual volume reached 5000 cases (^15^). Other countries, such as the United Kingdom and France, report 800 and 1600 adrenalectomies per year, respectively (^16^,^17^).

In 2005, a survey of 3382 computed tomography scans at a university hospital in southern Brazil (Porto Alegre, Rio Grande do Sul) identified adrenal incidentalomas in 2.5% of cases (^18^), a prevalence similar to the 2.3% reported in a 2003 autopsy series review by Barzon and cols. (^19^). This similarity may suggest a more conservative clinical approach in Brazil or perhaps a lower clinical priority given to such findings in the SUS routine, potentially resulting in patients who require specific procedures not being adequately evaluated or investigated.

### Surgical volume

Favorable outcomes have been associated with increased annual numbers of adrenalectomies, such as reduced perioperative mortality in high-volume centers (0.4%-0.56% versus 1.00%-1.25%) (^17^,^20^). Although these differences may appear modest, they become significant when compared to the aggregated perioperative mortality of bariatric surgery, estimated at around 0.08% (^21^).

Studies are heterogeneous regarding the volume threshold, and there remains controversy about whether the ideal responsible agent is the individual surgeon or the referral center as a whole. While advantages have been reported with surgeon expertise (^5^), hospitals equipped with multidisciplinary teams can provide the comprehensive assessment required for the complexity of adrenal diseases. The recommendation of the European Society of Endocrine Surgeons adopted the convergence point of current evidence (^5^,^16^), stating that adrenalectomies should be referred to hospitals performing at least six surgeries per year and that the treatment of adrenocortical carcinoma be restricted to those with a minimum annual volume of twelve cases (^22^).

As DATASUS did not provide information on which surgeon performed each procedure, we adopted the European Society of Endocrine Surgeons criteria for outcome analysis. The 26 high-volume hospitals performed 62.3% of the surgeries, despite oncology patients being more likely to be treated in low-volume hospitals. Although the overall mortality rate decreased during the study period and ICU admissions increased, hospitals with an annual volume above six adrenalectomies per year maintained a lower mortality rate compared to those with lower volumes. However, a superior result was not demonstrated when using a cutoff point of seven or more cases per year.

In low-volume hospitals, non-oncological adrenalectomies were associated with a 6.6-fold higher risk of in-hospital mortality compared to high-volume hospitals. For the non-association between surgical volume and mortality to be accepted (null hypothesis), it would require, beyond increased mortality in high-volume hospitals, a 55% reduction in mortality in hospitals performing fewer than six cases per year. Among oncological cases, mortality was 2.75 times higher in these same low-volume hospitals. Given that the number of oncological adrenalectomies in southern Brazil exceeds the global average incidence of the disease, adopting a reference threshold of twelve adrenalectomies per year for this region is advisable.

There were no significant differences between highand low-volume centers regarding length of hospital stay or ICU admissions. Nevertheless, among patients aged over 60 years who died, there was an association with treatment in low-volume hospitals. The possibility that comorbidities may have influenced outcomes should be considered (^23^).

### Geographic distribution

Of the twenty-six high-volume hospitals, twenty-four are located in the southern and southeastern regions, where the number of hospitalizations consistently exceeded the number of resident patients. This pattern suggests these hospitals received referrals from other regions, likely central-western Brazil, where the opposite trend was observed.

### Oncological adrenalectomies

Analysis of the data showed that patients who underwent unilateral or bilateral adrenalectomy did not do so for oncological purposes. If they had been, procedures would have been classified as oncological adrenalectomy, and the attending physician would have requested the corresponding code in the hospital admission authorization. Considering this, there was a substantial increase in oncological adrenalectomies, while the number of cases operated for presumably benign diseases remained stable. It is possible that increased detection of tumors with phenotypic warning signs (>4 cm, invasive features, multiple hormonal hypersecretion) contributed to this trend. The emergence of targeted therapies and immunotherapies for solid tumors may have also influenced the rise in adrenalectomies for metastatic lesions over the years (^24^,^25^). Another possibility is improved classification of adrenal tumors during the study period, resulting in more accurate coding for oncological cases.

It was not possible to extract information regarding hormonal status, the diagnosis (International Statistical Classification of Diseases and Related Health Problems 10th Revision) associated with the performed adrenalectomy, or the anatomopathological findings. Such information would provide important insights into the epidemiology of adrenal diseases and allow for cross-referencing of variables.

Another factor that may have contributed to the 6.44% reduction in non-oncological adrenalectomies was the restriction of 3 million elective surgeries in Brazilian public hospitals due to resource reallocation during the COVID-19 pandemic since 2019 (^26^). For instance, elective cholecystectomies decreased by 51% within the SUS system (^27^). In contrast, the last period of the study showed a 42.87% increase in oncological adrenalectomies. One hypothesis is that classification bias may have occurred, whereby the oncological code was used to facilitate elective procedures during the pandemic. Furthermore, the average reimbursement for oncological procedures was 188% higher than that for benign unilateral adrenalectomies.

Regarding age distribution among patients undergoing oncological adrenalectomy, a bimodal incidence pattern was observed, consistent with reports from other locations (^28^). An initial increase was noted in children aged 1-4 years, followed by another in adults aged 54-65 years. Similarly, in the United States, the average age at diagnosis is 55 years, with a predominance among white women (^29^). In the Brazilian cohort from 2018 to 2022, the incidence of oncological adrenalectomy per one million inhabitants in southern Brazil (2.04-2.72) was higher than the estimated global average of the disease (1.00-2.00) and that of the United States (0.72) (^30^,^31^).

Notably, the survey was based on the procedure code, which does not necessarily correspond to the definitive anatomopathological diagnosis of adrenal carcinoma or metastasis. Therefore, the 3000 cases of oncological adrenalectomy identified should be interpreted with caution. Nonetheless, a previous study from southern and southeastern Brazil reported a high incidence of adrenocortical tumors in children, 10-15 times greater than the global incidence (^32^,^33^), with germline TP53 mutations present in 50%-65% of cases (^34^). International data indicate that children typically present with the disease within the first 5 years of life, with a strong female predominance and almost universal virilization (^35^). However, in the 1-4 year age group, the female-to-male sex ratio was lower than previously reported (1.25:1 versus 1.6:1) (^32^).

Furthermore, we must consider that oncological adrenalectomies may exhibit higher in-hospital mortality rates due to increased severity and surgical complexity, including malignant pheochromocytoma cases within this group. Our analysis confirmed that mortality was higher compared to non-oncological cases. There is no data in Datasus regarding clinical comorbidities, intraoperative complications, rates of conversion to laparotomy, or reinterventions, which precluded full adjustment of the groups for multivariate logistic regression analysis. Therefore, the risk of postoperative death attributed to oncological surgery may have been overestimated.

### Surgical access routes

The inability to identify the surgical access route constitutes a limitation of this study, as there is no specific procedure code for laparoscopic adrenalectomy, unlike for cholecystectomy (^27^). According to published series from the cohort period, various access techniques were likely used (^36^-^38^).

Nevertheless, indications for open surgery as the primary approach are well established (^2^,^39^). Adrenocortical carcinomas are often diagnosed at a larger size than benign tumors at presentation. Tumors larger than 6 cm carry a 19% risk of malignancy, increasing to 47% for those larger than 8 cm (^40^). Therefore, it is likely that most oncological adrenalectomies were conducted via open surgery, as it is considered the best treatment in resectable cases (^41^). Another indirect indication supporting the predominance of this technique is the proportion of cholecystectomies performed by open approach in the SUS, estimated at 58.5% (^27^). Compared to other countries, the adoption of laparoscopic techniques for adrenalectomy varies significantly, reported heterogeneously at 78% in Scandinavia (^42^) and 35% in the United States (^20^).

In conclusion, the number of adrenalectomies performed in Brazil has increased, although it remains less common than in other countries. In the coming years, public health expenditures on the surgical treatment of adrenal diseases are expected to rise. The data collected confirmed the hypothesis that most adrenalectomies are unilateral, with an increasing proportion of oncological adrenalectomies especially among residents of states in southern Brazil, suggesting a regional incidence above the global average.

Despite the limitations of the DATASUS platform, indicators from this database have previously been analyzed and shown to be consistent regarding the registration of procedures (^27^). We consider the hospital mortality rate in our study to be reliable, as it represents dichotomous data and reflects the most impactful outcome, particularly when compared with the surgical volume of the participating hospitals. Optimal treatment of adrenal tumors is achieved when patients are referred to centers performing six or more adrenalectomies per year, which is associated with reduced in-hospital mortality.

For a research agenda, we propose: 1) Implement in DATASUS a procedure code for laparoscopic adrenalectomy and include the diagnosis (International Statistical Classification of Diseases and Related Health Problems 10th Revision) associated with adrenalectomy and the corresponding anatomopathological results; 2) Direct public health policies should be developed for Brazilian regions with a higher incidence of adrenalectomy for oncologic indications; 3) A national long-term follow-up database should be developed for the treatment of adrenocortical carcinoma, including anatomopathological staging and adjuvant treatments; 4) In five years, reassess whether the increase in the number of oncological adrenalectomies persists or whether the observed trend in this cohort was due to classification bias.
